# Identification of cadaveric liver tissues using thanatotranscriptome biomarkers

**DOI:** 10.1038/s41598-020-63727-9

**Published:** 2020-04-20

**Authors:** Gulnaz T. Javan, Erin Hanson, Sheree J. Finley, Silvia D. Visonà, Antonio Osculati, Jack Ballantyne

**Affiliations:** 1 Forensic Science Program, Physical Sciences Department, Alabama State University, Montgomery, AL USA; 2 National Center for Forensic Science, University of Central Florida, Orlando, FL USA; 3 Department of Public Health, Experimental and Forensic Medicine, University of Pavia, Pavia, Italy

**Keywords:** RNA sequencing, Transcriptomics

## Abstract

Thanatotranscriptome studies involve the examination of mRNA transcript abundance and gene expression patterns in the internal organs of deceased humans. Postmortem gene expression is indicative of the cellular status of a corpse at the time of death, a portion of which may represent a cascade of molecular events occasioned by death. Specific gene biomarkers identify perceptible transcriptional changes induced by stochastic responses to the cessation of biological functions. Transcriptome analyses of postmortem mRNA from a tissue fragment may determine unique molecular identifiers for specific organs and demonstrate unique patterns of gene expression that can provide essential contextual anatomical information. We evaluated the impact of targeted transcriptome analysis using RNA sequencing to reveal global changes in postmortem gene expression in liver tissues from 27 Italian and United States corpses: 3.5-hour-old to 37-day-old. We found that our single blind study using eight liver tissue-specific gene biomarkers (e.g. *AMBP* and *AHSG*) is highly specific, with autopsy-derived organ samples correctly identified as tissues originating from postmortem livers. The results demonstrate that 98–100% of sequencing reads were mapped to these liver biomarkers. Our findings indicate that gene expression signatures of mRNA exposed up to 37 days of autolysis, can be used to validate the putative identity of tissue fragments.

## Introduction

Emerging research to clarify the postmortem fingerprint of mRNA transcript expression patterns of the 25,000 human genes^1,2^ have been studied to clarify the secrets of how human beings die. Integrated approaches to investigate temporal variations and the eventual cessation of postmortem transcription expression patterns have influenced an upsurge in studies of the functional complexities of the thanatotranscriptome^3–8^. The thanatotranscriptome is derived from the Greek word for death (*thanatos*-), and it encompasses all RNA transcripts expressed from the part of genome that is still functional or that becomes awakened in internal organs of a dead body^3^. Before death, terminally differentiated cells in organs have unique patterns of gene expression. Gene expression is primarily regulated at the level of transcription by varying the production of mRNA from functional genes. Postmortem specificity of expressed genes during the destruction of cells involves broad and actively coordinated biological responses in a tissue-specific manner.

Previous studies have been performed using mRNA-based approaches to supplement traditional protein-based methods of identifying organ tissue sources^5,8^. The integrity of postmortem tissues samples is of paramount importance in regard to experiments that attempt to monitor gene expression at the precise stage of RNA extraction. RNA quality is contingent on tissue source; for example, liver and spleen are more abundant in ubiquitous RNases that degrade RNA molecules more rapidly and with a higher activity than less RNase-rich tissues (e.g. heart and muscle)^4,9,10^. Appropriate covariates such as the time elapsed since the initiation of postmortem autolysis^11^ and pH^12^ have been implicated as indicators of overall RNA quality and/or mRNA transcript abundances.

The ability to ascertain and interpret distinct transcriptional patterns are important determinations that are useful in a variety of areas. Studies have been conducted on organ transplantation^13–17^, organs-on-chips for drug discovery^15,16^, regenerative medicine (e.g., liver regeneration)^13,17^, and a plethora of rapidly evolving areas of crime scene investigations (e.g. traumatic injury)^18^. Therefore, it will be of interest to investigate these areas for more information, since they could provide understandings into how to best maintain the integrity of organs retrieved for these postmortem applications.

Many medicolegal cases consist of substantial injury to the human body’s internal organs which may be relocated from the injured body to another person or position^18^. For example, a gunshot injury caused by bullet compression or stretch may result in major tissue damage and adherence of organ tissue to bullets and/or projectiles. Allocated tissues depend on the occurrences of the crime and may involve the transference of different internal organs (e.g., brain, heart, liver). Tissue identification can be combined with standard forensic DNA analyses, and the differentiation and positive identification of biological specimens have the potential to be significant evidential information.

The primary aim of the current study is to validate the use of an MPS-based organ tissue identification assay at varying times of death using actual criminal casework. The study builds upon previous findings of the thanatotranscriptome that described targeted RNA expression analyses by ten commercially acquired organ tissue types using a targeted panel of 46 mRNA biomarkers^5^. This assay precisely identified tissue source origins using a single blind study of RNA mixtures from different internal organs. The results demonstrated the ability to successfully identify tissue sources without cross-reactions. In the present study, we hypothesized that as a human body decays, mRNA profiles will permit organ identification and biomarkers validation obtained from commercial liver tissues versus autopsy-derived sources. The liver was chosen for this study because it has the highest postmortem microbial taxon abundance^19^ which is attributable to several abiotic and biotic factors^20–22^. To test this hypothesis, we extracted total RNA and prioritized our approach by performing targeted transcriptome analysis using RNA-Seq in liver tissues obtained at autopsy from criminal casework cadavers in Italy and the United States. Italian cadavers were chosen for this study due to their extended PMIs compared to those of American cadavers. The Italian Regolamento di Polizia Mortuaria, Law number 285, Article 8 of 1990 dictates that medicolegal autopsies cannot be performed prior to 24 hours after discovery of the body^23^.

## Materials and Methods

### Preparation of postmortem liver samples

**Figure 1:**
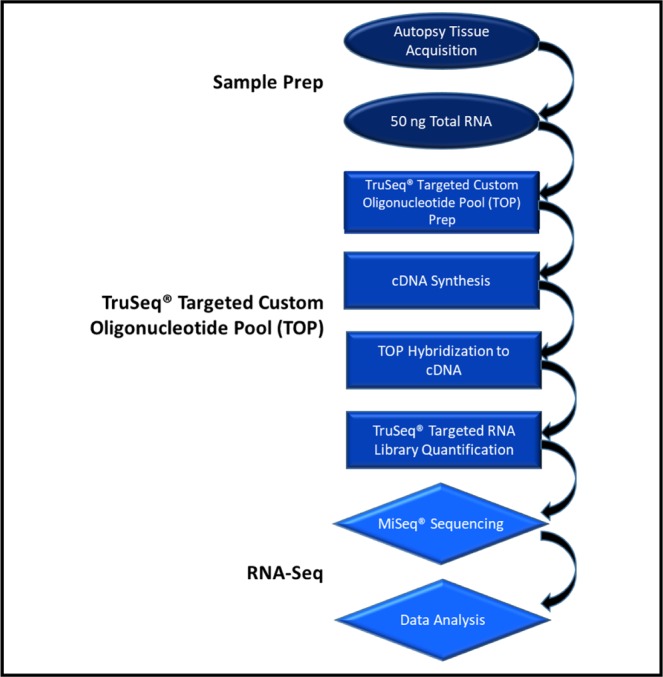
Thanatotranscriptome tissue identification assay. The assay involves sample preparation of liver tissue from criminal casework cadavers followed by RNA extraction. RNA was transcribed into first strand complementary DNA. A targeted RNA massively parallel sequencing (MPS) multiplex of 46 genes was prepared using the TruSeq Targeted RNA kit (Illumina Inc.) and a TruSeq Targeted RNA custom oligonucleotide pool designed using Illumina Design Studio. The custom TOP was hybridized to the cDNA. RNA was sequenced using Illumina MiSeq sequencing protocols. MiSeq sequencing software was used to analyze the data.

The targeted thanatotranscriptome tissue identification assay (Fig. 1) was performed using postmortem liver tissues from corpses under criminal review, 20 from the University of Pavia, Italy and 10 from forensic pathologists in Montgomery, AL and Pensacola, FL, USA. The criminal casework cadavers were kept in the morgues at 1 °C. The shortest postmortem interval (PMI) was 3.5 hours from the United States and the longest was 37 days from Italy. Also, cadavers corresponding to different manners of death were grouped into four categories: accidental death, homicide, natural death, and suicide (Table 1). Tissues were collected using protocols approved by Alabama State University’s Institutional Review Board (2018400). Pursuant to Florida code Title XXIX chapter 406.11(2)(b) and Alabama code 45–2–61.04 laws for studies outside of normal autopsies, signed consent forms from next of kin were obtained by the appropriate district attorney prior to the collection of the criminal casework tissues. Autopsies took place in laboratory conditions with a temperature between 2–4 °C. Postmortem samples were isolated using sterile, disposable surgical scalpels. Samples were placed into labeled polyethylene bags and stored at −80 °C until they were shipped on dry ice to the Thanatos Laboratory at Alabama State University in Montgomery, AL. Upon arrival, the samples were immediately stored at −80 °C until further analysis.

**Table 1:** Metadata for the 20 criminal case cadavers from the University of Pavia in Pavia, Italy and ten from forensic pathologists in Montgomery, AL and Pensacola, FL, USA. Cadavers corresponded to various causes of death and were grouped into four different categories of manner of death: accidental death, homicide, natural death, and suicide.

United States									
Case Number	Case	Age	Sex	Ethnicity	Height (cm)	Weight (kg)	PMI (hrs)	Cause of Death	Manner of Death
Case-1	Case G2	53	M	Caucasian	180	77	3.5	Gunshot Wound Chest	Suicide
Case-2	Case 37	55	M	Caucasian	195	112	6	Coronary Heart Disease	Natural Death
Case-3	Case G34	26	M	Caucasian	185	63	9	Gunshot Wound Head	Suicide
Case-4	Case G17	40	F	Hispanic	156	96	13	Combined Drug Intoxication	Accident
Case-5	Case G32	35	M	Caucasian	175	88	15	Gunshot Wound Head	Suicide
Case-6	Case 20	19	M	African American	178	80	17	Gunshot Wound	Homicide
Case-7	Case 9	19	M	Caucasian	191	177	20.5	Car Accident	Accident
Case-8	Case 26	23	M	African American	193	72	22	Multiple Gunshot Wound	Homicide
Case-9	Case 32	45	F	African American	178	117	29.5	Overdose	Accident
Case-10	Case G36	26	M	African American	175	127	32	Terminal Seizure	Natural Death
**Italy**									
Case-1	Italy 18	73	M	Caucasian	177	70	38	Thoracic Trauma	Accident
Case-2	Italy 3	79	M	Caucasian	171	80	42	Thoracic Trauma	Accident
Case-3	Italy 21	64	M	Caucasian	178	75	50	Thoracic Trauma	Accident
Case-4	Italy 13	53	M	Caucasian	178	90	72	Acute Myocardial Infarction	Natural Death
Case-5	Italy 41	40	M	Caucasian	175	90	80	Subarachnoid Hemorrhage	Accident
Case-6	Italy 43	67	F	Caucasian	157	120	96	Pneumonia	Natural Death
Case-7	Italy 32	92	M	Caucasian	167	50	120	Pneumonia	Natural Death
Case-8	Italy 34	53	M	Caucasian	176	75	48	Ischemic Heart Disease	Natural Death
Case-9	Italy 5	52	F	Caucasian	157	60	65	Burning Injuries	Suicide
Case-10	Italy 6	46	F	Caucasian	162	45	888	Respiratory Failure	Natural Death
Case-11	Italy 17	60	M	Caucasian	175	90	60	Cranic and Thoracic Injuries	Accident
Case-12	Italy 33	70	M	Caucasian	178	97	60	Ischemic Heart Disease	Natural Death
Case-13	Italy 12	80	F	Caucasian	158	50	144	Acute Heart Failure	Natural Death
Case-14	Italy44	85	F	Caucasian	162	90	96	Multiple Organ Failure	Natural Death
Case-15	Italy 45	36	F	Hispanic	156	60	240	Postpartum Hemorrhage	Natural Death
Case-16	Italy 42	67	M	Caucasian	174	75	144	Subarachnoid Hemorrhage	Accident
Case-17	Italy 31	63	M	Caucasian	159	60	72	Asphyxia	Accident
Case-18	Italy 24	41	M	Caucasian	179	75	168	Acute Drug Intoxication	Suicide
Case-19	Italy 40	33	M	Caucasian	166	80	108	Hemorrhagic Shock	Accident
Case-20	Italy 47	61	M	Caucasian	170	100	168	Heart Failure	Natural Death

### Total rna extraction and quantitation

To ensure the veracity of the extracted RNA, benchtops and instruments were cleaned with RNAase AWAY (Thermo Fisher Scientific) and allowed to dry before the start of extractions. Sterile disposable surgical scalpels (Thermo Fisher Scientific) were used to remove approximately 30 mg of liver tissues from each specimen as previous described^6^. Briefly, postmortem liver tissues were immediately deposited into 2 ml Lysing Matrix E tubes (MP Biomedicals) containing 1.4 mm ceramic spheres, 0.1 mm silica spheres, and one 4 mm glass bead. Total RNA from each sample was extracted in triplicates. RNeasy Lysis Buffer (RLT) (Qiagen) was added to the tubes for a total volume of 600 µl. To release nucleic acids within the hepatocytes, tubes were placed in a homogenizer for 5 min to disrupt over 90% of the liver cells. After washing the homogenized lysates 70% ethanol, and RNA was pipetted onto the RNeasy mini spin columns that were supplied in the RNeasy Isolation Kit (Qiagen). RNeasy spin columns were centrifuged and washed three times in ethanol. The columns were then washed with Buffer RW1 (Qiagen) and washed with Buffer RPE (Qiagen). The columns were washed lastly with Buffer RPE (Qiagen). After decanting supernatants, pellets were washed with 100 μl of ethanol and allow to dry at room temperature. RNA was eluted to 40 µl followed by Nanodrop analysis was used to measure concentrations. Quant-iTTM RiboGreen RNA Kit (Thermo Fisher Scientific) was used to quantitate RNA in the extracts and the fluorescence was determined using a SynergyTM 2 Multi-Mode microplate reader (BioTek Instruments, Inc.,). Extracts were then stored at −20 °C until the next analysis.

### TruSeq targeted RNA library preparation

As previously described, a targeted RNA deep sequencing that included a massively parallel sequencing (MPS) multiplex of 46 genes was employed^5^. Briefly, custom sequencing panels were prepared using the TruSeq Targeted RNA Expression kit (Illumina, Inc.), and the Illumina DesignStudio was used to design a custom TruSeq Targeted oligonucleotide pool (TOP). This custom panel included adipose, brain, heart, intestine, kidney, liver, lung, muscle, stomach and trachea biomarkers. *AMBP, F2, SPP2, CFHR2, F9, MBL2, AHSG, and C9* (Table 2) were the eight liver gene biomarkers included in the study. Thermal cycler reactions with extremely fast heating and cooling rates were performed using the Mastercycler Pro S Thermal Cycler (Eppendorf) and loaded in thin-walled skirted Microseal PCR plates (Bio-Rad) that were sealed with Microseal A film (Bio-Rad). Purification reactions were completed in 0.8 mL 96-well storage plates (Thermo Fisher Scientific) and were sealed with Microseal B film.

**Table 2:** Eight liver genes used in the 46-plex TruSeq targeted RNA custom oligonucleotide pool assay. *AMBP, F2, SPP2, CFHR2, F9, MBL2, AHSG, and C9* were the eight liver gene biomarkers choses for the study.

Liver Gene	Function	Illumina Assay ID
*AMBP*	Encodes alpha-1-microglobulin/bikunin precursor	6846165
*F2*	Encodes coagulation factor II/ thrombin precursor	6834705
*SPP2*	Encodes a secreted phosphoprotein	6646626
*CFHR2*	Encodes complement factor H-related 2 proteins	6824671
*F9*	Encodes vitamin K-dependent coagulation factor IX	6813125
*MBL2*	Encodes a mannose-binding lectin	6748563
*AHSG*	Encodes alpha 2-HS glycoprotein	6842654
*C9*	Encodes the final component of the complement system	6711440

### cDNA synthesis

RNA molecules were reverse transcribed into first strand complementary DNA (cDNA) via the protocol for the TruSeq Targeted RNA Expression kit for intact total RNA. In a 48-well reaction plate, a 4 µL mixture of reverse transcription cDNA synthesis master mix (RCS1) (Illumina, Inc.) was combined with 1 µL of ProtoScript II reverse transcriptase (New England Biolabs, Inc.), and 50 ng total RNA. Reaction plates were centrifuged for 1 min. The reverse transcription conditions involved the following protocol: 25 °C for 5 min, 42 °C for 15 min, 95 °C for 10 min, and an infinite hold at 4 °C.

### TruSeq Targeted RNA custom TOP library preparation

cDNA was hybridized to the custom TOP as previously described^5^. Briefly, the 10 µL reaction mixture contained 5 µL of the oligonucleotide pool (Illumina Inc.) and 5 µL of Tris-ETDA buffer (pH 8.0) (Thermo Fisher Scientific). Reaction plates were incubated at room temperature for 1 min then 30 µL of streptavidin magnet beads (OB1) (Illumina, Inc.) were added. The total volume of the reaction mixtures was 50 µl. The hybridization conditions involved the following protocol: 70 °C for 5 min, 68 °C for 1 min, 65 °C for 2.5 min, 60 °C for 2.5 min, 55 °C for 4 min, 50 °C for 4 min, 45**°**C for 4 min, 40**°**C for 4 min, 35 °C for 4 min, 30 °C for 4 min, and a hold at 30 °C. Following assay oligonucleotide hybridization, several wash steps are performed according to the manufacturer’s protocol. After adapters are ligated to nucleic acid fragments and extended, Index 1 (i7) adapters and Index 2 (i5) adapters were added to reaction mixtures. In the next amplification reaction, 20 µl of the purified extension–ligation products were added 50 µL of the amplification reaction then centrifuged for 1 min. The amplification involved the following protocol: 95 °C for 2 min, 34 cycles of 98 °C for 30 sec, 62 °C for 30 sec, 72 °C for 60 sec, 72 °C for 5 min, and an infinite hold at 10 °C. The final sample library volume was 12.5 µl. According to the manufacture’s recommendations, 5 µl of each sample library were combined in each sequencing reaction. The libraries were then measured on the TapeStation 2200 (Agilent Technologies) with High Sensitivity D1000 Screen tape. Average sizes were determined in nM for the 1/10 diluted libraries obtained from the 100–300 bp region.

### RNA sequencing

Denatured libraries containing 5 µL of the 4 nM were combined with 5 µL of 0.2 N NaOH then incubated at 25 °C for 5 min. Then, pre-chilled HT1 buffer (Illumina Inc.) was mixed with denatured library pools for final concentrations of 20 pM. 600 µL of 6 pM samples were pipetted into the MiSeq v3 150 cycle reagent cartridge (Illumina, Inc.) for sequencing on the MiSeq instrument which involved 51 single-end sequencing cycles. Samples were demultiplexed using the Illumina MiSeq control software (v.2.6.2.1) which is the pre-installed package on the MiSeq sequencing system. This software provides an overview of quality statistics monitored as the run progresses.

### Bioinformatic analyses

Local MiSeq sequencing software was used to perform analyze base calling, demultiplexing, and alignment utilizing the banded Smith-Waterman algorithm. The results of these analyses produced a target hits file that revealed the total reads per amplicon per sample. A total read count (MTR) of a minimum of 5000 was used as the threshold for each sample. Samples with counts below 5000 MTR were omitted from further analysis. A minimum biomarker read count (MBR) of 500 was used as the threshold for each sample. Samples with counts less than 500 MBR were omitted. Specific biomarker read count quantities that were below 0.5% of the total reads for the sample were also excluded. After filtering the liver samples, raw total read count data were visualized using scatter plots for each liver gene and bar graphs were generated to determine raw counts by trial and by liver gene biomarker. The percentage of total reads was uniquely calculated for each sample using biomarker read count divided by total count for the sample.

## Results

### Specificity of read counts of 46 gene biomarkers

A minimum sample total read count of 5000 was used as the sample threshold, and there were three postmortem liver cases that were below this threshold and were therefore excluded from further analysis. Between the 27 cadavers tested that were above this threshold, the average read count for liver biomarkers ranged from 93,387.5 (*AMBP*, encodes alpha-1-microglobulin/bikunin precursor) to 8,312 (*MBL2*, encodes mannose binding lectin 2) in Italian cases and 15,300.5 (*AMBP*) to 1,033.6 (*MBL2*) in United States cases (Tables S1 and S2, respectively). For Italian cases, expression was not observed for brain, lung, trachea, heart, kidney, intestine, and stomach biomarkers. Expression of *MBL2* was observed in only four of the 17 Italian liver samples (Table S1). Further, two Italian cases (48 hr and 65 hr) demonstrated expression of skeletal muscle biomarkers (*TNNI2* and *ATP2A1*, respectively) and three cases (42 hr, 50 hr, and 80 hr) showed expressions of the *PLIN1* adipose biomarker. For United States cases, expression was not observed for brain, lung, trachea, skeletal muscle, heart, kidney, and adipose biomarkers (Table S2). Unexpectedly, a liver biomarker, *AHSG*, did not demonstrate expression. Further, one American case (32 hr) demonstrated expression of two intestine biomarkers (*DEFA6* and *LCT*) and another American case (9 hr) showed expression of one stomach biomarker (*PGA3*).

### Percent contributions of reads

**Figure 2:**
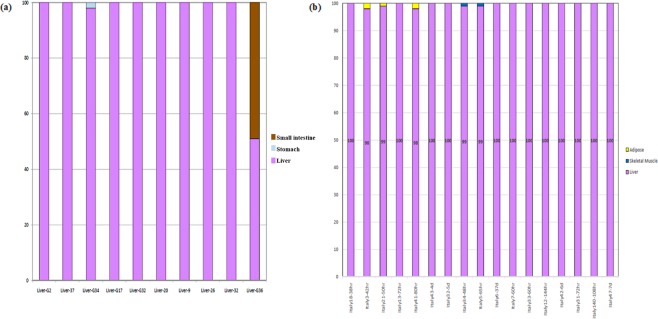
Expression of biomarker composition for individual liver samples for (**a**) Italian cases and (**b**) United States cases. The percent contribution to the sample for the liver biomarker was calculated (reads per liver biomarker/total reads per sample). The percentages from each biomarker were pooled into classes. A contribution of 100% indicates that all reads for the sample were attributable to liver. In the Italian cases, percent reads were also attributable to small intestine and stomach biomarkers. In United States cases, percent reads were attributable to adipose and skeletal muscle biomarkers.

Percent contribution of reads were calculated to provide the ratio of total reads for each sample that was attributable to liver-specific biomarkers. Read count data were filtered by threshold values and visualized using bar graphs plotting the percent contribution of representative liver gene biomarkers versus real postmortem liver tissues from cadaver cases from Italy and the United States (Fig. 2a,b, respectively). The results demonstrated that in each of the 27 cadavers, tissues were correctly attributable as postmortem liver samples. Some samples were identified to contain gene biomarkers for other tissues. However, overall, 98–100% of the reads were imputable to liver biomarkers (e.g., *AMBP*, *AHSG*).

**Figure 3:**
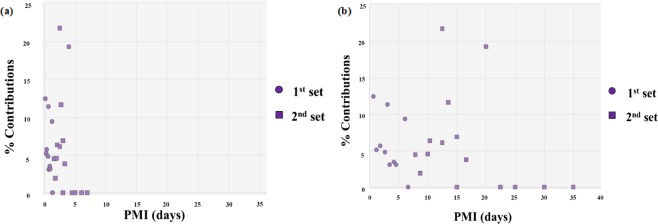
Scatter plot analysis of normalized gene expression of F2 (coagulation factor II) in human liver samples taken at various postmortem intervals (PMIs) and the TruSeq Targeted Expression Process. The percent contributions (marker read counts/sample count * 100) (**a**) PMIs up to 1 week and (**b**) the full panel of PMIs. Circles represent the American liver tissue samples analyzed with times of death within hours to one day. Squares represent the Italian liver tissue samples analyzed with longer times of death up to 37 days.

Some of the gene candidates were specifically chosen for use in changes in their abundance with different timeframes. For example, changes in the normalized expression (individual gene read count/total sample reads * 100) of the *F2* (coagulation factor II/thrombin precursor) gene in postmortem liver samples demonstrated that no expression of this gene is observed after approximately four days (Fig. 3).

## Discussion

The implementation of the MPS-based organ tissue identification assay was conducted using 30 total RNA liver samples derived from actual autopsy corpses. Due to low threshold values (<5000 total read counts), three of the Italian cases (Case 6, PMI 96 hrs; Case 15, PMI 240 hrs; and Case 20, PMI 168 hrs) were excluded from analyses. The results provide proof-of-principle of this biomarker assay to successfully verifying liver as the tissue source origin from cadaver tissues.

While the gene biomarkers were not specifically chosen for use in evaluating changes in transcript abundance, a few of the candidates exhibited shifts in their abundances with time of death. For example, changes in the normalized expression of the *F2* (coagulation factor II, thrombin) gene were demonstrated in postmortem liver samples (Fig. 3). No expression of this gene is observed after approximately four days postmortem. These findings confirmed the potential to determine the extent to which the anatomical site influences the combinatorial expression of liver biomarkers.

A preponderance of trauma casework involves the transfer of internal organ tissue from the decedent to other individuals, proximate areas of the crime scene, or suspected criminal implements. In some circumstances, the source of the organ tissue is undetermined. Novel, advanced molecular methods are required to update current approaches that still generally rely on time consuming and sometimes unsuccessful determinations of organ tissue sources made by microscopic histological analysis by pathology personnel. In addition to using forensic techniques to discriminate DNA evidence, an MPS-based thanatotranscriptome assay that can definitively identify human internal organ tissue could potentially provide contextual evidence about the source of the organ tissue present at the crime scene. The thanatotranscriptome has recently been advocated as an innovative molecular approach in forensic science, specifically to identify the source of organ tissue and to determine the manner of death^3,5,6,24^.

The liver was chosen for this proof-of-principle study due to it being a large, forensically relevant organ in the abdominal cavity that has the highest postmortem microbial taxon abundance^19^. Due to its size and location, a bullet or knife penetration in the front of the abdomen has a high likelihood of damaging the liver and a piece thereof being transferred to the weapon or projectile. It should also prove useful in subsequent studies for PMI estimations because of time-dependent microbiota-induced RNA degradation. Multiple internal organ tissues beyond the liver will be the subject of subsequent double-blind studies.

RNA methods involving next generation sequencing have been recently developed to increase the capabilities of forensic identification of biological specimens. In a related study involving whole miRNome massively parallel sequencing, partial least squares (PLS) and linear discriminant analyses (LDA) were used to screen six forensically relevant bodily fluids and/or tissues in a data set of 119 specimens^25^. A subclass of RNA biomarkers, miRNAs, were used in the study. miRNAs are small (18–24 nucleotides), non-coding RNAs that are involved in RNA silencing and post-transcriptional regulation of gene expression^25^. The results demonstrated successful prediction of body fluids on the basis of the expression of miRNA markers. In another recent developmental validation study, ParaDNA Body Fluid ID System was tested to identify body fluids in field-deployable protocols^26^. The system detected and analyzed mRNA from six body fluids and concluded that the Body Fluid ID System can effectively determine the presence of body fluid mRNA biomarkers in both single-source and mixed specimens from multiple substrate types. Further, as little as 0.05 ng of total RNA and one μl of relevant body fluids could be identified using this novel technique^26^.

In conclusion, postmortem gene expression was analyzed by high-throughput “omics” techniques, which are powerful approaches to transcriptome profiling that can evaluate the importance of decomposition across PMI and cause of death. For this research, it was established that RNA molecules are stable in postmortem liver samples up to seven days which makes RNA a suitable molecule for gene expression studies. The study design validates a technique that will meet the demand for rapid and reproducible thanatotranscriptomic methods. The current study is the first to use the MPS methodology with RNA sequencing methods to identify internal organ tissue from actual criminal cases. Future studies will include the analysis of mixed tissue organ samples to train and test our RNA-Seq-based tissue identification multiplex model accordingly. Our targeted MPS analysis could be incorporated into forensic kits and enhance existing methods to identify organ tissue sources.

## Supplementary information

Supplementary Tables.

## References

[1] Yeh RF, Lim LP, Burge CB (2001). Computational inference of homologous gene structures in the human genome. Genome Res..

[2] Trenchevska O, Phillips DA, Nelson RW, Nedelkov D (2014). Delineation of concentration ranges and longitudinal changes of human plasma protein variants. PLoS One.

[3] Javan GT, Can I, Finley SJ, Soni S (2015). The apoptotic thanatotranscriptome associated with the liver of cadavers. Forensic Sci. Med. Pathol..

[4] Walker DG (2016). Characterization of RNA isolated from eighteen different human tissues: results from a rapid human autopsy program. Cell Tissue Bank..

[5] Hanson E, Ballantyne J (2017). Human organ tissue identification by targeted RNA deep sequencing to aid the investigation of traumatic injury. Genes (Basel)..

[6] Tolbert M (2018). The thanatotranscriptome: Gene expression of male reproductive organs after death. Gene.

[7] Ferreira PG (2018). The effects of death and post-mortem cold ischemia on human tissue transcriptomes. Nat. Commun..

[8] Salzmann AP, Russo G, Aluri S, Haas C (2019). Transcription and microbial profiling of body fluids using a massively parallel sequencing approach. Forensic Sci. Int. Genet..

[9] Fleige S (2006). Comparison of relative mRNA quantification models and the impact of RNA integrity in quantitative real-time RT-PCR. Biotechnol. Lett..

[10] Bauer M (2007). RNA in forensic science. Forensic Sci. Int. Genet..

[11] Castensson A, Emilsson L, Preece P, Jazin E (2000). High-resolution quantification of specific mRNA levels in human brain autopsies and biopsies. Genome Res..

[12] Kingsbury AE (1995). Tissue pH as an indicator of mRNA preservation in human post-mortem brain. Mol. Brain Res..

[13] Giwa S (2017). The promise of organ and tissue preservation to transform medicine. Nat. Biotechnol..

[14] Prakash SK (2019). “Donating our bodies to science”: A discussion about autopsy and organ donation in Turner syndrome. Am. J. Med. Genet. C Semin. Med. Genet..

[15] Esch EW, Bahinski A, Huh D (2015). Organs-on-chips at the frontiers of drug discovery. Nat. Rev. Drug Discov..

[16] Jodat YA (2018). Human-Derived Organ-on-a-Chip for Personalized Drug Development. Curr. Pharm. Des..

[17] Kren BT, Steer CJ (1996). Posttranscriptional regulation of gene expression in liver regeneration: role of mRNA stability. FASEB J..

[18] DiMaio, V.J. Introduction to the classification of gunshot wounds. In *Gunshot wounds* 80-131 (CRC Press, 2015).

[19] Javan GT (2016). Human thanatomicrobiome succession and time since death. Scientific Rep..

[20] Javan GT (2019). An interdisciplinary review of the thanatomicrobiome in human decomposition. Forensic Sci. Med. Pathol..

[21] Finley SJ, Pechal JL, Benbow ME, Robertson BK, Javan GT (2016). Microbial signatures of cadaver gravesoil during decomposition. Microb. Ecol..

[22] Jordan HR, Tomberlin JK (2017). Abiotic and biotic factors regulating inter-kingdom engagement between insects and microbe activity on vertebrate remains. Insects.

[23] Frati P (2006). Neuroanatomy and cadaver dissection in Italy: history, medicolegal issues, and neurosurgical perspectives: Historical vignette. J. Neurosurg..

[24] Sampaio-Silva F, Magalhães T, Carvalho F, Dinis-Oliveira RJ, Silvestre R (2013). Profiling of RNA degradation for estimation of post morterm interval. PLoS One.

[25] Dørum G (2018). Predicting the origin of stains from next generation sequencing mRNA data. Forensic Sci. Int. Genet..

[26] Blackman S (2018). Developmental validation of the ParaDNA Body Fluid ID System—A rapid multiplex mRNA-profiling system for the forensic identification of body fluids. Forensic Sci Int Genet..

