# Supplementary material for: Identification of cadaveric liver tissues using thanatotranscriptome biomarkers
Source: Sci Rep. 2020 Apr 20;10:6639. doi: 10.1038/s41598-020-63727-9 (PMC7170907; doi:10.1038/s41598-020-63727-9)
Supplement: Supplementary file 1 — Supplementary Tables. [file 41598_2020_63727_MOESM1_ESM.pdf]

Identification of cadaveric liver tissues using thanatotranscriptome biomarkers

Gulnaz T. Javan[1*], Erin Hanson[2], Sheree J. Finley[1], Silvia D. Visonà[3], Antonio Osculati[3], Jack Ballantyne[2]

[1]Forensic Science Program, Physical Sciences Department, Alabama State University, Montgomery, AL

[2]National Center for Forensic Science, University of Central Florida, Orlando, FL

[3]Department of Public Health, Experimental and Forensic Medicine, University of Pavia, Pavia, Italy

***Corresponding Author**
Alabama State University
Physical Sciences Department
Forensic Science Programs
915 S. Jackson Street
Montgomery, AL 36104
Email: gjavan@alasu.edu
Phone: 334-604-8130

Table S1. Tissue Specificity of Read Counts of 46 Gene Biomarkers in Italian Cases

	Postmortem Interval (PMI)																	
	**Gene Name**	**38hr**	**42hr**	**48hr**	**50hr**	**60hr**	**60hr**	**65hr**	**72hr**	**72hr**	**80hr**	**4d**	**108hr**	**5d**	**144hr**	**6d**	**7d**	**37d**
**Brain**	**SNAP25**	0	0	0	0	0	0	0	0	0	0	0	0	0	0	0	0	0
	**RTN1**	0	0	0	0	0	0	0	0	0	0	0	0	0	0	0	0	0
	**GABRA1**	0	0	0	0	0	0	0	0	0	0	0	0	0	0	0	0	0
	**OPALIN**	0	0	0	0	0	0	0	0	0	0	0	0	0	0	0	0	0
	**GFAP**	0	0	0	0	0	0	0	0	0	0	0	0	0	0	0	0	0
	**NEUROD6**	0	0	0	0	0	0	0	0	0	0	0	0	0	0	0	0	0
**Lung**	**SFTPB**	0	0	0	0	0	0	0	0	0	0	0	0	0	0	0	0	0
	**SFTPD**	0	0	0	0	0	0	0	0	0	0	0	0	0	0	0	0	0
	**SFTPA1**	0	0	0	0	0	0	0	0	0	0	0	0	0	0	0	0	0
**Trachea**	**BPIFB1**	0	0	0	0	0	0	0	0	0	0	0	0	0	0	0	0	0
**Liver**	**AMBP**	**107441**	**68337**	**93613**	**675279**	**16121**	**24766**	**161803**	**178151**	**5824**	**104827**	**30196**	**10568**	**6404**	**33700**	**8913**	0	**61645**
	**F2**	**34146**	**6360**	**32084**	**147187**	**27338**	**6079**	**72986**	**75618**	0	**15209**	**19054**	0	0	0	0	0	0
	**CFHR2**	**15520**	**2154**	**10796**	**20488**	0	**4432**	**11209**	0	0	**3231**	**3757**	0	0	0	0	0	0
	**F9**	0	0	**5857**	**23521**	**989**	0	**7381**	0	0	0	0	**6984**	0	0	0	0	**6032**
	**AHSG**	**524477**	**202367**	**473793**	**1284572**	**81272**	**49107**	**353684**	**831523**	**542**	**246640**	**32828**	**93624**	**10885**	**40898**	0	**51560**	**254777**
	**C9**	**41351**	**19764**	**58902**	**35359**	0	**15476**	0	0	0	**8167**	**13178**	0	0	0	0	0	**24466**
	**SPP2**	**18082**	**3982**	**8327**	**36191**	0	0	**11913**	**12283**	**2603**	**18070**	0	**1341**	0	0	0	0	0
	**MBL2**	**24361**	**23876**	**14268**	**78799**	0	0	0	0	0	0	0	0	0	0	0	0	0
**Skeletal Muscle**	**TNNI2**	0	0	8158	0	0	0	0	0	0	0	0	0	0	0	0	0	0
	**MYLK2**	0	0	0	0	0	0	0	0	0	0	0	0	0	0	0	0	0
	**ATP2A1**	0	0	0	0	0	0	9206	0	0	0	0	0	0	0	0	0	0
	**MYH2**	0	0	0	0	0	0	0	0	0	0	0	0	0	0	0	0	0
	**NEB**	0	0	0	0	0	0	0	0	0	0	0	0	0	0	0	0	0
	**MYLPF**	0	0	0	0	0	0	0	0	0	0	0	0	0	0	0	0	0
**Heart**	**ITGB1BP3**	0	0	0	0	0	0	0	0	0	0	0	0	0	0	0	0	0
	**MYBPC3**	0	0	0	0	0	0	0	0	0	0	0	0	0	0	0	0	0
	**NPPB**	0	0	0	0	0	0	0	0	0	0	0	0	0	0	0	0	0
	**NPPA**	0	0	0	0	0	0	0	0	0	0	0	0	0	0	0	0	0
	**TNNI3**	0	0	0	0	0	0	0	0	0	0	0	0	0	0	0	0	0
**Kidney**	**UMOD**	0	0	0	0	0	0	0	0	0	0	0	0	0	0	0	0	0
	**SLC12A1**	0	0	0	0	0	0	0	0	0	0	0	0	0	0	0	0	0
	**SLC34A1**	0	0	0	0	0	0	0	0	0	0	0	0	0	0	0	0	0
	**SLC22A12**	0	0	0	0	0	0	0	0	0	0	0	0	0	0	0	0	0
**Adipose**	**TUSC5**	0	0	0	0	0	0	0	0	0	0	0	0	0	0	0	0	0
	**ADIPOQ**	0	0	0	0	0	0	0	0	0	0	0	0	0	0	0	0	0
	**PLIN1**	0	6412	0	17621	0	0	0	0	0	6127	0	0	0	0	0	0	0
**Intestine**	**FABP6**	0	0	0	0	0	0	0	0	0	0	0	0	0	0	0	0	0
	**CCL25**	0	0	0	0	0	0	0	0	0	0	0	0	0	0	0	0	0
	**DEFA5**	0	0	0	0	0	0	0	0	0	0	0	0	0	0	0	0	0
	**DEFA6**	0	0	0	0	0	0	0	0	0	0	0	0	0	0	0	0	0
	**LCT**	0	0	0	0	0	0	0	0	0	0	0	0	0	0	0	0	0
**Stomach**	**PGA3**	0	0	0	0	0	0	0	0	0	0	0	0	0	0	0	0	0
	**PGA4**	0	0	0	0	0	0	0	0	0	0	0	0	0	0	0	0	0
	**GIF**	0	0	0	0	0	0	0	0	0	0	0	0	0	0	0	0	0
	**GKN1**	0	0	0	0	0	0	0	0	0	0	0	0	0	0	0	0	0
	**PGA5**	0	0	0	0	0	0	0	0	0	0	0	0	0	0	0	0	0
	**Average Total**	**765,378**	**333,252**	**705,798**	**2,319,017**	**125,720**	**99,860**	**628,182**	**1,097,575**	**8,969**	**402,271**	**99,013**	**112,517**	**17,289**	**74,598**	**8,913**	**51,560**	**346,920**

Average read counts of liver biomarker in Italian liver tissue samples (calculated from N=17 cases). For each donor liver tissue, the average total read counts are listed. Only samples with total read counts greater than 5000 were analyzed. Average read counts in red indicate read counts for biomarkers representing tissues from other organs.

Table S2. Tissue Specificity of Read Counts of 46 Gene Biomarkers in United States Cases

		Postmortem Interval (PMI)									
	**Gene Name**	**3.5hr**	**6hr**	**9hr**	**13hr**	**15hr**	**17hr**	**20.5hr**	**22hr**	**29.5hr**	**32hr**
**Brain**	**SNAP25**	0	0	0	0	0	0	0	0	0	0
	**RTN1**	0	0	0	0	0	0	0	0	0	0
	**GABRA1**	0	0	0	0	0	0	0	0	0	0
	**OPALIN**	0	0	0	0	0	0	0	0	0	0
	**GFAP**	0	0	0	0	0	0	0	0	0	0
	**NEUROD6**	0	0	0	0	0	0	0	0	0	0
**Lung**	**SFTPB**	0	0	0	0	0	0	0	0	0	0
	**SFTPD**	0	0	0	0	0	0	0	0	0	0
	**SFTPA1**	0	0	0	0	0	0	0	0	0	0
**Trachea**	**BPIFB1**	0	0	0	0	0	0	0	0	0	0
**Liver**	**AMBP**	**3400**	**23637**	**9619**	**17204**	**4083**	**28850**	**24170**	**26111**	**11409**	**4522**
	**F2**	**885**	**2906**	**1627**	**2239**	**1904**	**3029**	**3209**	**1425**	**2148**	
	**CFHR2**	0	0	0	**514**	0	**1677**	**2340**	0	0	0
	**F9**	0	**632**	0	0	0	**839**	**1536**	0	0	0
	**AHSG**	0	0	0	0	0	0	0	0	0	0
	**C9**	0	**1824**	**1888**	**1427**	0	**3498**	**3505**	**992**	**513**	0
	**SPP2**	**2843**	**27518**	**11314**	**22050**	**9967**	**59891**	**56872**	**16416**	**8132**	**5444**
	**MBL2**	0	0	**3569**	**3127**	**838**	0	**541**	**774**	**686**	**801**
**Skeletal Muscle**	**TNNI2**	0	0	0	0	0	0	0	0	0	0
	**MYLK2**	0	0	0	0	0	0	0	0	0	0
	**ATP2A1**	0	0	0	0	0	0	0	0	0	0
	**MYH2**	0	0	0	0	0	0	0	0	0	0
	**NEB**	0	0	0	0	0	0	0	0	0	0
	**MYLPF**	0	0	0	0	0	0	0	0	0	0
**Heart**	**ITGB1BP3**	0	0	0	0	0	0	0	0	0	0
	**MYBPC3**	0	0	0	0	0	0	0	0	0	0
	**NPPB**	0	0	0	0	0	0	0	0	0	0
	**NPPA**	0	0	0	0	0	0	0	0	0	0
	**TNNI3**	0	0	0	0	0	0	0	0	0	0
**Kidney**	**UMOD**	0	0	0	0	0	0	0	0	0	0
	**SLC12A1**	0	0	0	0	0	0	0	0	0	0
	**SLC34A1**	0	0	0	0	0	0	0	0	0	0
	**SLC22A12**	0	0	0	0	0	0	0	0	0	0
**Adipose**	**TUSC5**	0	0	0	0	0	0	0	0	0	0
	**ADIPOQ**	0	0	0	0	0	0	0	0	0	0
	**PLIN1**	0	0	0	0	0	0	0	0	0	0
**Intestine**	**FABP6**	0	0	0	0	0	0	0	0	0	0
	**CCL25**	0	0	0	0	0	0	0	0	0	0
	**DEFA5**	0	0	0	0	0	0	0	0	0	0
	**DEFA6**	0	0	0	0	0	0	0	0	0	6169
	**LCT**	0	0	0	0	0	0	0	0	0	4042
**Stomach**	**PGA3**	0	0	598	0	0	0	0	0	0	0
	**PGA4**	0	0	0	0	0	0	0	0	0	0
	**GIF**	0	0	0	0	0	0	0	0	0	0
	**GKN1**	0	0	0	0	0	0	0	0	0	0
	**PGA5**	0	0	0	0	0	0	0	0	0	0
	**Average Total**	7,128	56,517	28,615	46,561	16,792	97,784	92,173	45,718	22,888	20,978

Average read counts of liver biomarker in American liver tissue samples (calculated from N=10 donors). For each donor liver tissue, the average total read counts are listed. Only samples with total read counts greater than 5000 were analyzed. Average read counts in red indicate read counts for biomarkers representing tissues from other organs.
